# Direct and crossover effects of Phenylephrine and Cyclopentolate on foveal avascular zone and vessel density of macular capillary plexuses: an optical coherence tomography angiography study

**Published:** 2020

**Authors:** Yasin Şakir Göker, Hasan Kızıltoprak, Kemal Tekin, Esat Yetkin, Mustafa Salih Karatepe, Kübra Özdemir, Gokhan Demir

**Affiliations:** * Ulucanlar Eye Training and Research Hospital, Ankara, Turkey; ** Ercis State Hospital, Ophthalmology Department, Van, Turkey; *** Fatih Sultan Mehmet Training and Research Hospital, Istanbul, Turkey

**Keywords:** capillaries, mydriatics, optical coherence tomography

## Abstract

**Purpose:** To determine the influence of phenylephrine and cyclopentolate on foveal avascular zone (FAZ) and vessel density of macular capillary plexus measurements via optical coherence tomography angiography (OCTA).

**Materials and Methods:** The participants were separated into 2 groups according to the administration of drops. One drop of phenylephrine 2.5% was administered on one eye of each subject in the phenylephrine group (n=30) and one drop of cyclopentolate 1% in the cyclopentolate group (n=30). FAZ parameters and vessel density values of both superficial (SCP) and deep capillary plexuses (DCP) were calculated via OCTA priorly and at 45 min following the drop administration in both eyes. Vessel density, acircularity index of FAZ, FAZ area, perimeter of FAZ and foveal density-300 were evaluated via OCTA.

**Results:** The vessel density values of fovea in SCP and DCP was 18.51±7.14% and 36.05±8.76% and significantly decreased to 16.16±6.26% and 33.29±9.47% respectively after drop instillation in dilated eyes in phenylephrine group (p=0.041 and p=0.032). Likewise, the vessel density values in SCP and DCP were 21.56±7.74% and 39.27±8.76% and significantly decreased to 18.89±7.32% and 35.36±5.75% respectively, after drop instillation in dilated eyes in cyclopentolate group (p=0.035 and p=0.028). However, there was no significant difference between before and after instillation of drops in terms of foveal density-300 value via FAZ assessment tool in both dilated and nondilated contralateral eyes in both groups (p>0.05 for all).

**Conclusions:** Mydriasis with phenylephrine and/ or cyclopentolate did not affect the foveal density-300 values while analyzing the perfusion of macula. Vessel density in foveal region should be evaluated via FAZ evaluation software of the OCTA.

## Introduction

Over the last years, optical coherence tomography angiography (OCTA) has become an important imaging technique in terms of differential diagnosis of macular disorders including senile macular degeneration, type 3 neovascularization, perifoveal telangiectasia and retinal polyps [**1**-**3**]. It is a safe and advanced resolution imaging modality that allows the assessment of retinal microcirculation without the need of fluorescein usage [**4**]. OCTA also allows the evaluation of foveal avascular zone (FAZ) features, capillary nonperfusion areas, vessel density in both superficial (SCP) and deep capillary plexuses (DCP) quantitatively in different retinal disorders [**5**-**8**]. While evaluating vessel density in foveal region, different algorithms of OCTA including density evaluation software and/ or FAZ evaluation software could be used [**5**].

Phenylephrine and cyclopentolate are two commonly used mydriatics in clinical practice. Phenylephrine, an alpha agonist, implements its mydriatic action by stimulating the iris dilator muscle and cyclopentolate, a parasympatholytic agent, which obtains pupil dilatation by limiting iris sphincter muscle activity. Moreover, cyclopentolate has a cycloplegic and long acting effect. Systemic effects of topical ocular medication mostly occur after absorption through the nasal mucosa or from the anterior segment of the eye. Both sympathomimetics and parasympatholytics have vasoconstricting effects and they may affect the measurements of the OCTA parameters. The objective of this present study was to find out the effect of phenylephrine and cyclopentolate on FAZ area and capillary density of foveal region measurements in healthy subjects using FAZ evaluation software, non-flow evaluation software and density evaluation software of OCTA.

## Materials and Methods

This cross-sectional study with prospective enrollment was carried out at Ulucanlar Eye Research and Training Hospital. Written and oral informed consent were provided from all the participants. The study was approved by the Ethical Committee of Numune Training and Research Hospital (21.03.2018/ 1788) and adhered to the Declaration of Helsinki.

**Examination protocol and screening**

All the participants had an entire ophthalmic evaluation, including evaluation of best corrected visual acuity (BCVA), biomicroscopic examination of anterior segment, gonioscopy with Goldman three mirror lens and applanation tonometry. Axial length (AL) calculations, central corneal thickness (CCT) and OCTA measurements were also performed.

**Eligibility criteria**

Inclusion criteria were BCVA better than or equal to 20/ 20, less than 2 diopters of spherical or cylindrical refractive error, nonsmoking, no drug therapy, no alcohol use, nonexistence of glaucomatous evidence and intraocular pressure (IOP) levels over 21 mmHg. The exclusion criteria were anterior segment obscurity, AL < 21 millimeters and > 24 millimeters, and history of ocular intervention within 12 months before the study entry. Individuals with pathological findings (such as retinal vascular abnormalities, iridocyclitis, or vitreoretinal interface disorders) were excluded. Patients with diabetes mellitus and hypertension were also removed from the study. 

**Study groups**

The individuals were randomly separated into 2 groups according to the application of drops as previously described by Kara and associates [**9**]:

**Phenylephrine group (Group 1).** The individuals took a drop of phenylephrine (Mydfrine®, Alcon, USA) 2.5% three times at 5 minutes span in one eye. 

**Cyclopentolate group (Group 2).** The individuals took a drop of cyclopentolate (Sikloplejin®, Abdi İbrahim, TR) 1% thrice at 5 minutes span in one eye.

The left eye was assigned the study eye for phenylephrine group individuals with an even-numbered birth year and the right eye was assigned the study eye for cyclopentolate group individuals with an odd-numbered birth year. Appropriate subjects were included in the study consecutively until group 1 and 2 achieved 30 individuals.

**OCTA calculations**

The same ophthalmologist performed the OCTA calculations using the via AngioVue software (Version 2017.1.0.151) of the RTVue XR Avanti (Opto-Vue, Inc, Fremont, CA) during the same period of the day (between 10-12 AM) and in aqua environmental situations. All images were of 6 mm × 6 mm scanning region centered on macula. One experienced independent observer assessed the angiography slabs. Individuals with low signal strength index (SSI <7) were excluded.

**Fig. 1:**
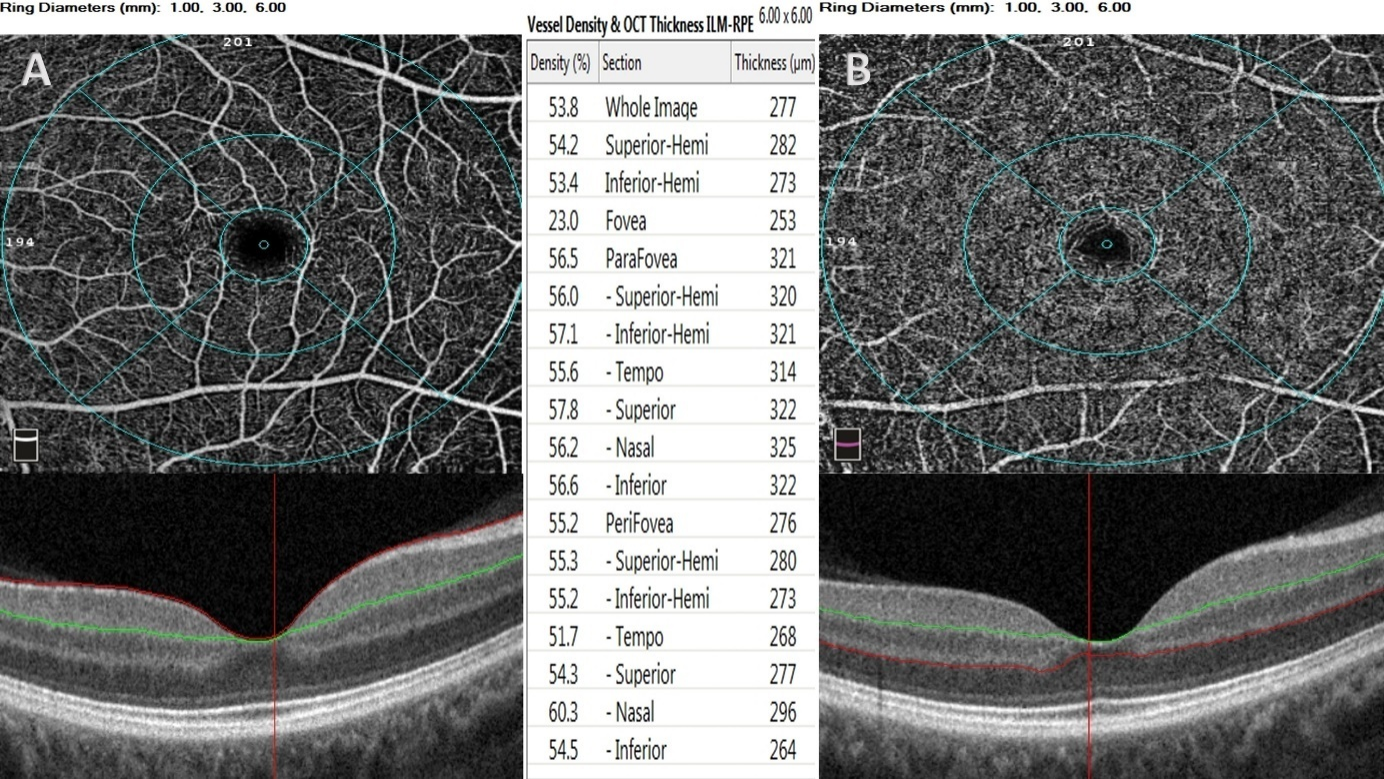
The device placed three fovea-centered rings on the macula by using density assessment software in both superficial capillary plexus (A) and deep capillary plexus (B) slabs. The zones were separated to equal parts

**Fig. 2:**
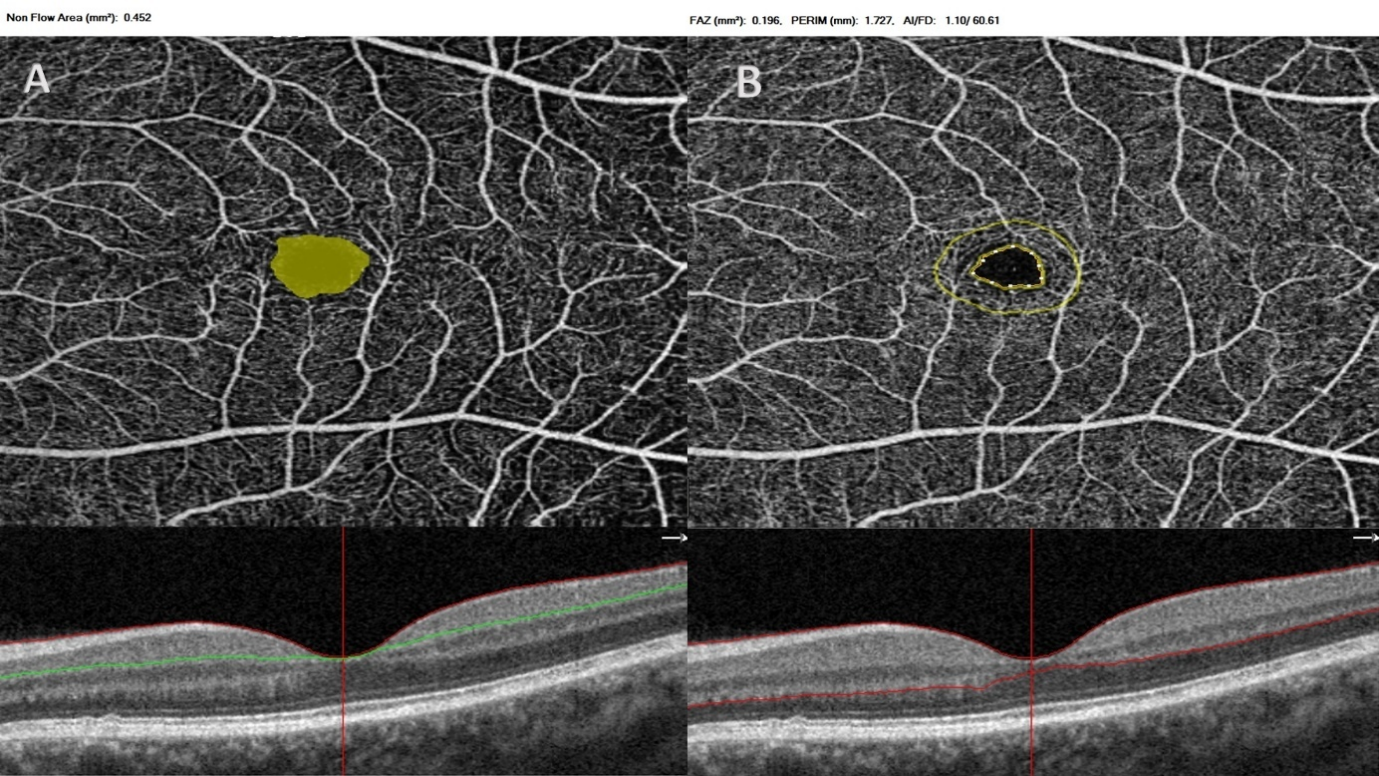
Non-flow FAZ area (mm2) in superficial capillary plexus was automatically provided using non-flow evaluation software (2A), and the FAZ area in whole retina (mm2), foveal density (%) (FD-300) (vessel density in 300 microns around the FAZ), FAZ perimeter (PERIM) (mm) and acircularity index (AI) of FAZ were also automatically provided using FAZ evaluation software (**Fig. 2B**)

The machine placed three fovea-centered rings on the macula by using density assessment software in both SCP and DCP slabs (**Fig. 1**). Foveal zone was described as 1 mm diameter circle area, the parafoveal zone was defined as 3 mm diameter middle circle area, and perifoveal zone was described as 6 mm diameter outer circle area. Additionally, the zones were separated into two equal hemispheres (inferior and superior). Non-flow FAZ area in SCP was automatically provided using non-flow evaluation software (**Fig. 2A**), and the FAZ area in whole retina, foveal density (FD-300) (vessel density in 300 microns around the FAZ), FAZ perimeter and acircularity index (AI) of FAZ were also automatically provided using FAZ evaluation software (**Fig. 2B**). 

**Statistical analysis**

SPSS (Statistical Package for the Social Sciences) (version 22 for Windows) was used for the statistical analysis. Kolmogorov–Smirnov test was used to evaluate the normality of the data (p > 0.05). Numerical variables were saved as mean and standard deviation and categorical data were saved as numbers. The categorical variables between the group 1 and 2 were evaluated via the χ2 test. The comparisons of the FAZ area values in superficial capillary plexus and whole retina, perimeter and AI of FAZ, FD-300 and vessel density values prior and following the administration of drops were analyzed with paired t-tests. A p value less than 0.05 was considered significant.

## Results

One hundred twenty eyes of 60 individuals were evaluated in this prospective study: 30 individuals in group 1 and 30 individuals in group 2, between December 2017 and June 2018. **Table 1** demonstrates the clinical characteristics and demographic data of the study individuals. The mean age in phenylephrine and cyclopentolate group was 42.10±16.72 (20-68) and 41.90±13.89 (20-66) years respectively. The mean axial length was 23.13±0.41 (22.20-23.5) mm in phenylephrine group and 23.20±0.33 (22.69-23.60) mm in cyclopentolate group. Spherical equivalent refraction was -0.14±0.98 (-2.0-2.0) diopters in phenylephrine group and 0.04±0.69 (-1.5-1.5) diopters in cyclopentolate group. The mean CCT and IOP values were 548±26.93 (505-600) µm and 14.43±2.16 (10-18) mmHg in phenylephrine group and 552±31.06 (497-624) µm and 15.60±3.10 (10-19) mmHg in cyclopentolate group respectively. Age, IOP, CCT, spherical equivalent refraction, AL and gender distribution did not show a significant difference between groups (P > 0.05 for all).

**Foveal Avascular Zone and Macular Capillary Plexus Vessel Density Changes**

The clinical data before and after instillation of drops in dilated eye and in contralateral eye are shown in **Tables 2**-**5**. The vessel density values of fovea via density evaluation software in SCP and DCP were 18.51±7.14% and 36.05±8.76% and significantly decreased to 16.16±6.26% and 33.29±9.47% respectively after drop instillation in dilated eyes in phenylephrine group (p=0.041 and p=0.032) (**Table 2**). The vessel density values in SCP and DCP were 21.56±7.74% and 39.27±8.76% and significantly decreased to 18.89±7.32% and 35.36±5.75% respectively after drop instillation in dilated eyes in cyclopentolate group (p=0.035 and p=0.028) (**Table 4**). However, this difference did not demonstrate a statistical significance in foveal region in nondilated contralateral eyes (p=0.104 and p=0.684 in group 1 and p=0.140 and p=0.284 in group 2 respectively) (**Tables 3**,**5**). On the other hand, the FD-300 value via FAZ assessment tool in full retinal vasculature was not affected in dilated eyes in both groups. It was 53.44±7.00 and 52.64±5.59 in phenylephrine and cyclopentolate group and decreased to 52.97±7.41 and 51.44±3.73 respectively but this difference did not demonstrate a significant difference (p=0.842 and p=0.884 respectively) (**Tables 2**,**4**). Moreover, perimeter of FAZ, AI of FAZ, vessel density values in parafoveal and perifoveal zones and FAZ area in full retinal vasculature and in SCP did not demonstrate a significant difference before and after instillation of drops in both dilated and non-dilated contralateral eyes (P > 0.05 for all) (**Tables 2**-**5**).

**Table 1:** Clinical and Demographic Characteristics of the Study Individuals

Variable	*Phenylephrine group*	*Cyclopentolate group*	P*
Age (years)			
Mean±SD	42.10±16.72	41.90±13.89	0.662*
Range	(20-68)	(20-66)	
Sex (n)			
Male (%)	13 (%43)	13 (%43)	0.196**
Female (%)	17 (%57)	17 (%57)	
CCT, µm			
Mean±SD	548±26.93	552±31.06	0.096*
Range	(505-600)	(497-624)	
Axial Length, mm			
Mean±SD	23.13±0.41	23.20±0.33	0.346*
Range	(22,20-23,5)	(22.69-23.60)	
Spherical Equivalent, D			
Mean±SD	-0.14±0.98	0.04±0.69	0.894*
Range	(-2.0-2.0)	(-1.5-1.5)	
IOP, mmHg			
Mean±SD	14.43±2.16	15.60±3.10	0.112*
Range	(10-18)	(10-19)	
*D = diopter; CCT = central corneal thickness; IOP = intraocular pressure; µm = micrometer; SD = standard deviation.*			
*: Independent samples test. **: χ2 test.			

**Table 2:** Phenylephrine group (Dilated eye)

	*Before *	*After*	*Difference*	P*
	Mean±SD	Mean±SD		
*FAZ area (mm2) in SCP*	0.57±0.26	0.57±0.26	0.00	0.984
*FAZ area (mm2) in Full Retinal Vasculature*	0.30±0.13	0.30±0.12	0.00	0.216
*Perimeter (mm)*	2.09±0.50	2.04±0.48	-0.05	0.146
*Acircularity index*	1.09±0.03	1.08±0.01	-0.01	0.104
*FD-300 (%)*	53.44±7.00	52.97±7.41	-0.47	0.842
*Vessel density in SCP (Flow)%*				
Whole Image	49.78±3.41	48.45±4.19	-1.33	0.234
Superior-Hemi	49.72±3.26	48.41±3.70	-1.31	0.220
Inferior-Hemi	49.85±3.61	48.49±4.70	-1.36	0.264
Fovea	18.51±7.14	16.16±6.26	-2.35	0.041
Parafovea	51.29±5.56	49.56±6.27	-1.73	0.541
Perifovea	50.78±3.25	49.66±4.32	-1.12	0.304
*Vessel density in DCP (Flow)%*				
Whole Image	51.04±6.60	47.85±7.59	-3.19	0.086
Superior-Hemi	50.97±6.81	47.52±7.55	-3.45	0.074
Inferior-Hemi	51.12±6.52	48.19±7.96	-2.93	0.122
Fovea	36.05±8.76	33.29±9,47	-2.76	0.032
Parafovea	55.34±5.90	53.92±6.87	-1.42	0.105
Perifovea	52.68±7.14	49.30±8.46	-3.38	0.108
*SD = Standard deviation; FAZ = Foveal avascular zone; FD = foveal density-300; SCP = superficial capillary plexus; DCP = deep capillary plexus.*				
*: Paired t-test				

**Table 3:** Phenylephrine group (Contralateral eye)

	*Before *	*After*	*Difference*	P*
	Mean±SD	Mean±SD		
*FAZ area (mm2) in SCP*	0.61±0.34	0.53±0.12	-0.08	0.232
*FAZ area (mm2) in Full Retinal Vasculature*	0.33±0.16	0.29±0.11	-0.04	0.154
*Perimeter (mm)*	2.20±0.56	2.07±0.42	-0.13	0.191
*Acircularity index*	1.10±0.03	1.09±0.02	-0.01	0.135
*FD-300 (%)*	52.04±4.74	52.43±4.22	0.39	0.470
*Vessel density in SCP (Flow)%*				
Whole Image	48.87±5.43	48.59±2.44	-0.28	0.212
Superior-Hemi	48.82±5.42	48.59±2.33	-0.23	0.211
Inferior-Hemi	48.93±5.51	48.60±2.67	-0.33	0.174
Fovea	19.27±6.45	17.97±6.53	-1.30	0.104
Parafovea	50.09±7.92	49.40±2.34	-0.69	0.745
Perifovea	49.88±5.05	49.02±2.12	-0.86	0.865
*Vessel density in DCP (Flow)%*				
Whole Image	49.86±8.13	48.74±5.35	-1.12	0.542
Superior-Hemi	50.11±8.05	49.49±5.40	-0.62	0.546
Inferior-Hemi	49.65±8.28	47.99±5.67	-1.66	0.528
Fovea	34.33±9.63	33.87±8.20	-0.46	0.684
Parafovea	54.94±6.97	54.66±4.06	-0.28	0.260
Perifovea	50.98±8.90	50.44±5.40	-0.54	0.393
*SD = Standard deviation; FAZ = Foveal avascular zone; FD = foveal density-300; SCP = superficial capillary plexus; DCP = deep capillary plexus.*				
*: Paired t-test				

**Table 4:** Cyclopentolate group (Dilated eye)

	*Before *	*After*	*Difference*	P*
	Mean±SD	Mean±SD		
*FAZ area (mm2) in SCP*	0.53±0,18	0.50±0.14	-0.03	0.240
*FAZ area (mm2) in Full Retinal Vasculature*	0.27±0.12	0.25±0.11	-0.02	0.091
*Perimeter (mm)*	1.99±0.51	1.90±0.45	-0.09	0.162
*Acircularity index*	1.09±0.03	1.08±0.04	-0.01	0.144
*FD-300 (%)*	52.64±5.59	51.44±3.73	-1.20	0.884
*Vessel density in SCP%*				
Whole Image	50.60±3.70	49.37±2.87	-1.23	0.186
Superior-Hemi	50.70±3.85	49.45±2.72	-1.25	0.554
Inferior-Hemi	50.46±3.77	49.30±3.12	-1.16	0.662
Fovea	21.56±7.74	18.89±7.32	-2.67	0.035
Parafovea	52.33±5.73	51.12±3.99	-1.21	0.126
Perifovea	51.50±3.73	50.19±2.87	-1.31	0.234
*Vessel density in DCP%*				
Whole Image	50.50±4.84	47.45±5.30	-3.05	0.146
Superior-Hemi	50.48±4.76	47.43±5.57	-3.05	0.651
Inferior-Hemi	50.53±5.13	47.47±5.21	-3.06	0.324
Fovea	39.27±6.10	35.36±5.75	-3.91	0.028
Parafovea	51.51±3.77	48.09±3.90	-3.42	0.670
Perifovea	52.27±5.28	49.54±6.12	-2,73	0.394
*SD = Standard deviation; FAZ = Foveal avascular zone; FD = foveal density-300; SCP = superficial capillary plexus; DCP = deep capillary plexus.*				
*: Paired t-test				

**Table 5:** Cyclopentolate group (Contralateral eye)

	*Before *	*After*	*Difference*	P*
	Mean±SD	Mean±SD		
*FAZ area (mm2) in SCP*	0.49±0.16	0.50±0,15	0.01	0.514
*FAZ area (mm2) in Full Retinal Vasculature*	0.26±0.09	0.26±0,10	0.00	0.511
*Perimeter (mm)*	1.96±0.39	1.98±0,45	0.02	0.756
*Acircularity index*	1.10±0.05	1.10±0,03	0.00	0.474
*FD-300 (%)*	54.47±4.48	53.92±5,57	0.55	0.660
*Vessel density in SCP%*				
Whole Image	50.83±3.54	50.73±2.69	-0.10	0.831
Superior-Hemi	50.89±3.32	50.78±2.34	-0.09	0.742
Inferior-Hemi	50.77±3.88	50.68±3.12	-0.09	0.901
Fovea	21.44±7.52	20.17±6.82	-1.27	0.140
Parafovea	53.09±3.79	52.93±4.30	-0.16	0.691
Perifovea	51.40±3.60	51.27±2.63	-0.13	0.635
*Vessel density in DCP%*				
Whole Image	51.55±6.71	51.40±5.27	-0.15	0.360
Superior-Hemi	51.63±6.75	51.36±5.34	-0.28	0.421
Inferior-Hemi	51.48±6.80	51.44±5.35	-0.04	0.324
Fovea	39.24±8.97	38.14±9.23	-1.10	0.284
Parafovea	55.80±4.55	55.74±3.68	-0.06	0.296
Perifovea	52.97±7.26	52.90±5.90	-0.07	0.346
*SD = Standard deviation; FAZ = Foveal avascular zone; FD = foveal density-300; SCP = superficial capillary plexus; DCP = deep capillary plexus.*				
*: Paired t-test				

## Discussion

Mydriatics are commonly used to dilate pupils for fundus examination for a lot of retinal circumstances and prior to ocular surgeries. Also, they were used prior to different imaging modalities including fundus fluorescein angiography, indocyanine green angiography and OCTA. Commonly used mydriatics are phenylephrine (a sympathomimetic), tropicamide and cyclopentolate (parasympatholytics). Phenylephrine induces vasoconstriction by its α1 adrenergic effect in peripheral capillaries including the conjunctival vessels and anterior ciliary arteries [**10**]. Tropicamide and cyclopentolate had no effect on peripheral vessels although they have muscarinic receptors on them. But, the main effects of these drugs on peripheral vessels were the decreased parasympathetic tone and the increased sympathetic tone. 

Mizuno et al. reported that the instillation of topical drugs can pass through the conjunctival pouch, through the periocular region and reach the retrobulbar region. In this region, the agent achieves an effective dose around the central retinal artery and short posterior ciliary arteries [**11**]. Moreover, after topical usage, the drug could diffuse into the globe and a direct effect could be observed through the retinal capillary [**11**,**12**]. In the macular region, the microvessels had a shortage of smooth muscle and they were managed by pericytes that were induced by adrenergic receptors [**13**-**15**].

OCTA allows clinicians to quantitatively measure different parameters including FAZ area, AI, FD-300 and vessel densities of macular capillary plexuses in various retinal disorders [**5**-**8**]. These parameters offer information about the prognosis of retinal diseases and can also be used in the follow-up process. Mydriasis is frequently an important step to provide a good retinal image. In this present study, we aimed to find out the effects of phenylephrine and cyclopentolate on the FAZ area and vessel density of macular capillary plexus measurements using density, non-flow and FAZ evaluation software of the OCTA.

In our study, FAZ evaluation software parameters including FAZ perimeter, acircularity index of FAZ, FAZ area and FD-300 did not change in both eyes of both groups (p>0.05). The FD-300 values in phenylephrine and cyclopentolate groups were 53.44±7.00% and 52.64±5.59% and changed to 52.97±7.41% and 51.44±3.73% respectively, after drop instillation in dilated eyes (p=0.842 and p=0.884). The mydriatics did not affect the FD-300 parameter. On the other hand, density values of SCP and DCP in foveal zone via density evaluation software of OCTA in both groups were significantly decreased (p=0.041 and p=0.032 in group 1 and p=0.035 and p=0.028 in group 2 respectively). Whole image density, parafoveal zone density and perifoveal zone density did not change significantly in both phenylephrine and cyclopentolate groups in dilated eyes (p>0.05 for all). 

Foveal density-300 is a vessel density variable of the FAZ evaluation software of OCTA. The instrument discovers the boundaries of avascular zone and draws a new ring around this zone at a space of 300 µ (**Fig. 2**) [**16**]. The instrument always calculates the vessel density in between these rings. However, in the vessel density evaluation software of OCTA, the rings are fixed at a diameter of 1, 3, and 6 mm in two different plexuses including SCP and DCP (**Fig. 1**) and the central 1 mm area includes lower vessel density values when compared with the FD-300 values. In the light of these findings, we speculated that the mydriatics did not affect the FD-300 values via the FAZ evaluation software of the OCTA in full retinal segmentation and we hypothesized that vessel density in foveal region should be evaluated via FAZ evaluation software of the OCTA.

Vessel density parameters did not show any significant changes in non-dilated eyes in both plexuses of both groups (p>0.05 for all). Topical usage of ophthalmic drugs could induce alteration in contralateral eye or might lead to systemic side effects [**17**-**19**]. Systemic effects of ocular drugs could be seen after the absorption through the cornea and conjunctiva or via the nasal mucosa. Also, eye drops could accumulate in the anterior chamber and could be disseminated to systemic circulation via the iridocorneal angle [**20**]. However, in our study we did not observe any significant alteration in the vessel density value of foveal region in contralateral eyes.

Cheng et al. evaluated the influence of topical mydriatic eye drops (0.5% tropicamide/ 0.5% phenylephrine mixture and 0.5% tropicamide alone) on the macular and peripapillary circulation via OCTA in eight healthy subjects [**21**]. They reported that vessel density of the peripapillary area was reduced, however, there was no significant reduction in the macular areas. The limitations of their study are the very small sample size and the fact that they evaluated the macula with the flow evaluation software of the device without dividing plexuses as SCP and DCP. Density evaluation software of the device allow clinicians to asses both SCP and DCP in different zones separately.

Our study has contributed as a recent invention to literature, highlighting the fact that the topical usage of phenylephrine and cyclopentolate did not affect the foveal density-300 values of macular region via FAZ assessment tool of OCTA in full retinal vasculature segmentation in dropped eyes and undropped contralateral eyes. The strength of the present study is its prospective design. Another strength is that measurements were made automatically using FAZ, density and non-flow evaluation software of the device. Previous studies demonstrated high reproducibility and reliability of these calculations [**22**-**25**]. We also explored the crossover effects of mydriatics that is another strength of our study. 

## Conclusion

Mydriasis with phenylephrine and/ or cyclopentolate did not affect the foveal density-300 values while analyzing the perfusion of macula. Vessel density in foveal region should be evaluated via FAZ assessment tool of the OCTA. 

**Acknowledgements**

The article has not been presented in any meeting.

**Sources of funding**

No funding was received for this research. 

**Conflict of interest**

All authors certify that they have no affiliations with or involvement in any organization or entity with any financial interest or non-financial interest in the subject matter or materials discussed in this manuscript. 
